# Systematic Profiling of Alternative Splicing Events in Ovarian Cancer

**DOI:** 10.3389/fonc.2021.622805

**Published:** 2021-03-08

**Authors:** Jia Liu, Dekang Lv, Xiaobin Wang, Ruicong Wang, Xiaodong Li

**Affiliations:** ^1^Department of Gynecology, Liaoning Cancer Hospital & Institute, Cancer Hospital of China Medical University, Shenyang, China; ^2^Cancer Center, Institute of Cancer Stem Cell, Dalian Medical University, Dalian, China; ^3^Department of Gynecology and Obsterics, First Affiliated Hospital of Dalian Medical University, Dalian, China

**Keywords:** ovarian cancer, alternative splicing, integrated prognostic model, LASSO, splicing factor

## Abstract

Alternative splicing (AS) is significantly related to the development of tumor and the clinical outcome of patients. In this study, our aim was to systematically analyze the survival-related AS signal in ovarian serous cystadenocarcinoma (OV) and estimate its prognostic validity in 48,049 AS events out of 21,854 genes. We studied 1,429 AS events out of 1,125 genes, which were significantly related to the overall survival (OS) in patients with OV. We established alternative splicing features on the basis of seven AS events and constructed a new comprehensive prognostic model. Kaplan-Meier curve analysis showed that seven AS characteristics and comprehensive prognostic models could strongly stratify patients with ovarian cancer and make them distinctive prognosis. ROC analysis from 0.781 to 0.888 showed that these models were highly efficient in distinguishing patient survival. We also verified the prognostic characteristics of these models in a testing cohort. In addition, uni-variate and multivariate Cox analysis showed that these models were superior independent risk factors for OS in patients with OV. Interestingly, AS events and splicing factor (SFs) networks revealed an important link between these prognostic alternative splicing genes and splicing factors. We also found that the comprehensive prognosis model signature had higher prediction ability than the mRNA signature. In summary, our study provided a possible prognostic prediction model for patients with OV and revealed the splicing network between AS and SFs, which could be used as a potential predictor and therapeutic target for patients with OV.

## Introduction

Ovarian cancer is one of the most common malignant tumors in women and the fifth leading cause of death among women with serious gynecological problems. It is estimated that there are 13,940 deaths and 21,750 new cases in the United States Cancer Statistics in 2020 (1). Due to the late diagnosis of ovarian cancer and the lack of effective treatment at present, the prognosis of OC patients is very poor, and the 5-year survival rate is only 30–40% (2). Although the diagnosis and treatment of OC have been improved to some extent in the past 30 years, OC is still a serious malignant tumor, threatening the lives of women (3). Therefore, the risk assessment of prognosis is of great value for clinical decision-making and patient consultation.

So far, due to the lack of high sensitivity and specificity, several common bio-markers for the diagnosis of OC are still not satisfactory, such as carbohydrate antigen 125 (CA125) (4) and human epididymal protein 4 (HE4) (5). At the same time, some studies have identified some genes that were significantly related to the prognosis of OC patients, such as TRIM44 (6) and CENPK (7). However, due to the inconsistency of sample collection, detection methods and sample size, the prognostic value of a single candidate index is very limited. Many reports have shown that, compared with a single bio-marker, integrated bio-markers can improve the accuracy of prognosis (8). Therefore, extensive studies have attempted to establish molecular characteristics based on gene expression data to predict the survival and prognosis of patients, including mRNA (9), microRNAs (3) and long non-coding RNA (LncRNA) based signatures (10). However, although these promising traits played an important role in predicting the survival of patients with OV, they mainly focused on changes in gene expression levels, ignoring the diversity of RNA sub-types regulated by alternative splicing (AS).

Alternative splicing, a post-transcriptional process by which a single pre-mRNA can be spliced into different arrangements to produce mRNA sub-types and protein diversity (11). Many studies have shown that this process has a great impact on the occurrence and development of cancer, including metastasis, therapeutic resistance, and other carcinogenic processes (12). In normal physiological processes, more than 95% of human genes have AS events and encode various splicing variants (13). More importantly, recent trends in different types of cancer research have shown that AS-related genes have new potential in cancer treatment (14). There is growing evidence that AS is associated with carcinogenic processes, including proliferation, metastasis, apoptosis, angiogenesis, hypoxia, and immune escape (15). In addition, previous studies showed that widespread dysfunctional AS events in a variety of cancers could be easily programmed by different SFs (16, 17). The overall changes may occur in some cancer-specific AS events resulting from the expression of these SFs changes, thus affecting the occurrence, and progression of cancer. Since the close relationship between AS and SFs is only understood from the surface of their complexity, it is of great significance to study their potential prognostic manifestations and regulatory mechanisms in OV. Therefore, it is very necessary to explore AS signature for the survival of OV patients.

Some studies have screened out some important AS and SF in ovarian cancer (18). However, their results were not obtained through strict scientific methods. In this study, univariate Cox, LASSO, and multivariate analysis were performed to systematically develop AS events related to prognosis in OV, and to establish a predictive model based on AS to evaluate the prognostic ability of AS signatures in patients with OV and to improve the understanding of tumor biology and oncology applications.

## Materials and Methods

### Data Collection and Processing

SpliceSeq data, RNA sequencing data and corresponding clinical information were downloaded from the TCGA database (https://tcga-data.nci.nih.gov/tcga/). A java application, SpliceSeq, was used to calculate the AS spectrum of each OV patient, which could unequivocally quantify the mRNA splicing level of the sample in TCGA. The Percent Spliced In (PSI) value was calculated to quantify AS events from 0 to 1, representing the frequency of different AS events. The validated data set contained 172 patients who were randomly selected from the overall population.

### Survival Analysis

A total of 344 OV patients participated in the study. All AS events were included and uni-variate analysis was performed. These AS events were reserved as candidate prognostic events (*P* < 0.05). Kaplan-Meier curve was used to evaluate the differential prognosis. Using the “Survival ROC” R packet, the receiver operating characteristic curve (ROC) was performed to explore the sensitivity and specificity of prognostic features. Through univariate and multivariate Cox analysis, forest map R packet was used to evaluate the prognostic independence and clinical characteristics of AS signatures.

### UpSet Plot and Splicing Factor to Regulate Network Construction

The Upset interaction plot was developed a more scalable visual diagram to explore the interactive set of these AS events and to use the “Upset” R package to visualize their potential interrelationships. The expression data of splicing factor (SFs) was extracted from TCGA-OV mRNA-seq data. All SF genes were analyzed by uni-variate Cox analysis. When their *P* < 0.05, these SF were considered to be survival-related splicing factors. The relationship between the expression value of SFs and the PSI value of AS was calculated by Spearman test. At the same time, Cytoscape 3.7.0 (https://cytoscape.org/) was used to illustrate the interactive network diagram of these SFs and prognosis-related AS events.

### Establishment of Prognostic Model

The least absolute shrinkage and selection operator (LASSO) analysis was carried out with “glmnet R” package, and the most valuable and concise AS events filtered in univariate Cox analysis were screened out (*P* < 0.05). Afterwards, the prognostic independence of AS signature was constructed by multivariate Cox analysis. Then, according to the coefficients of the above AS events, the risk score of each patient was calculated with the signature. At the same time, patients were divided into subgroups according to the median risk score.

### Statistical Analysis

*P*-value <0.05 was considered statistically significant. All analyses were performed using R 3.5.3 software (https://www.r-project.org/, v3.5.3).

## Result

### Overview of AS Events Analysis in TCGA-OV

**Figure 1:**
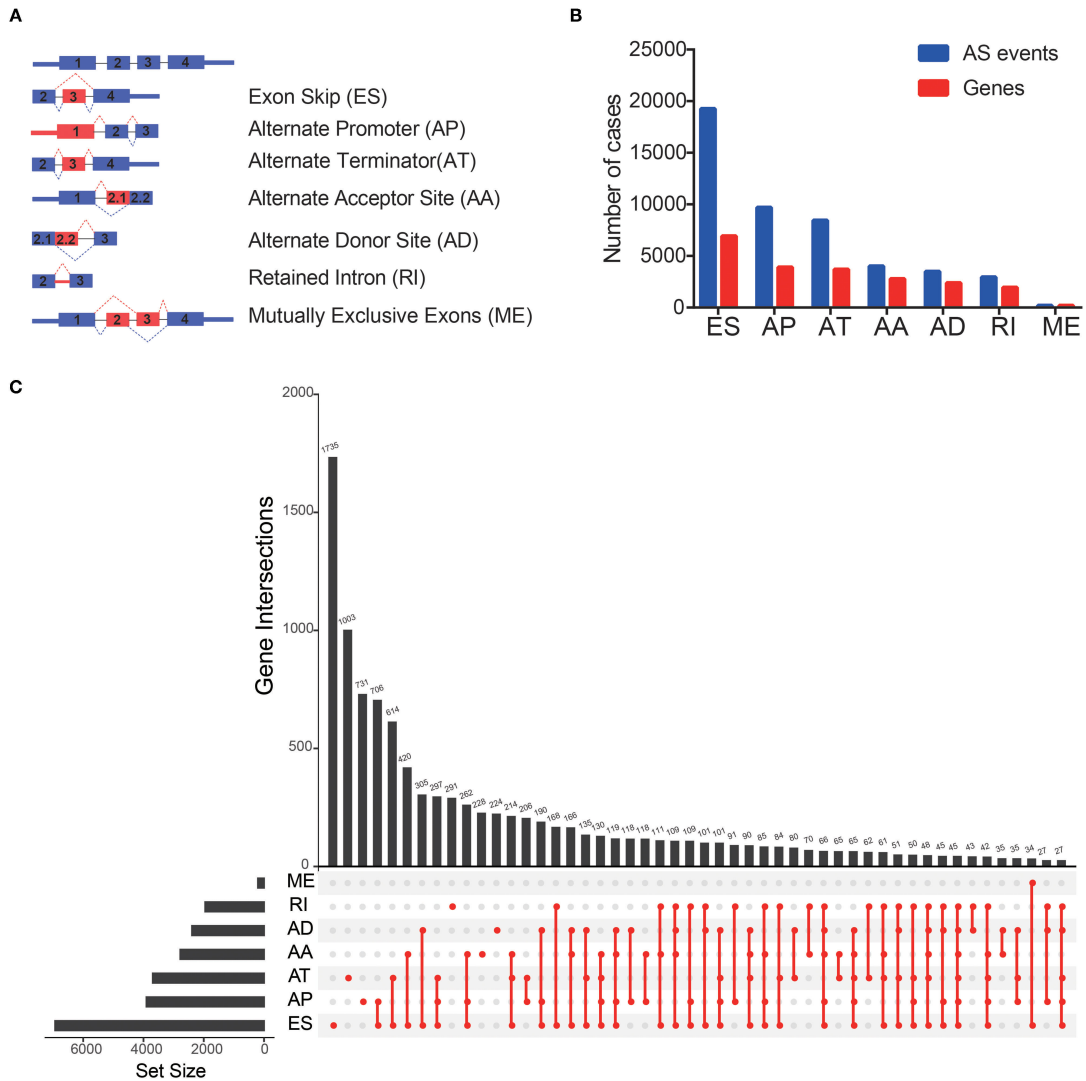
Overview of AS events in TCGA OV cohort. **(A)** Seven types of AS events were illustrated including exon skip (ES), retained intron (RI), alternate promoter (AP), alternate terminator (AT), alternate donor site (AD), alternate acceptor site (AA), and mutually exclusive exons (ME). **(B)** Numbers of AS events and AS-associated genes in 344 OV patients. **(C)** UpSet plot of overlapping genes among the seven patterns of AS events.

Comprehensive AS events were examined in 344 patients with OV (Figure 1A). A total of 48,049 AS events from 21,854 genes were detected, including 19,252 Exon Skip (ES) in 6,932 genes, 9,690 Alternate Promoter (AP) in 3,902 genes, 8,454 Alternative Promoter (AT) in 3,692 genes, 4,007 Alternate Acceptor (AA) in 2,778 genes, 3,498 Alternate Donor (AD) in 2,390 genes, 2,947 Retained Intron (RI) in 1,952 genes, 208 Mutually Exclusive Exons (ME) in 208 genes (Figure 1B). In TCGA-OV, ES events were the most common spliced signatures, accounting for more than 1/3 of all events, followed by AP and AT events, and ME was the least. It was worth noting that the number of AS events far exceeded their corresponding mRNAs. In addition, a subset of overlapping AS events in various types of AS in OV was shown by the UpSet diagram (Figure 1C).

### Development of Prognosis-Related AS Events in OV

**Figure 2:**
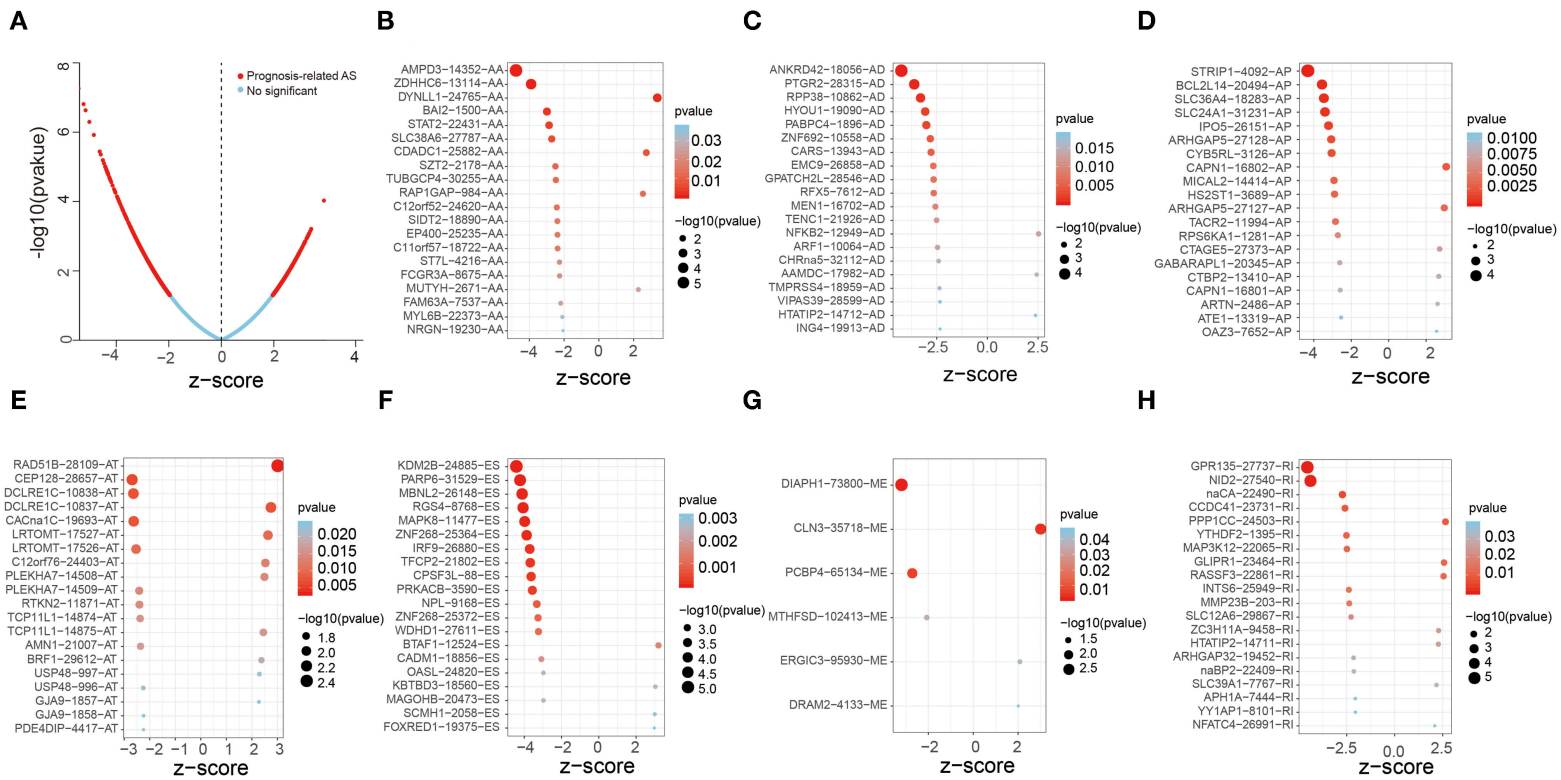
Forest plots analysis of survival-related AS events. **(A)** The Volcano plot depicts the *P*-values from the uni-variate Cox analysis of 48,049 AS events. **(B–H)** Forest plots of z-score of the top 20 significantly survival-related AS events for seven splicing types (ME has only six events).

In order to screen out the AS events related to the overall survival time of OV patients, 48,049 AS events were involved and univariate Cox analysis was performed. The results showed that 1,429 AS events out of 1,125 genes were significantly associated with overall survival in OV patients (Figure 2A). Figures 2B–H showed the top 20 significant prognosis-associated AS events of the seven types. Then Lasso Cox analysis was performed to further select the AS events related to the overall survival and prognosis of OV patients, which could reduce the coefficient and be designed as a linear regression background (19). Interestingly, some of these AS genes experienced multiple types of AS events. For example, AA, AD, ES of TMUB2 and AA, AT, ES of RBM39 were obviously related to overall survival.

### Construction of Prognostic AS Signatures

**Figure 3:**
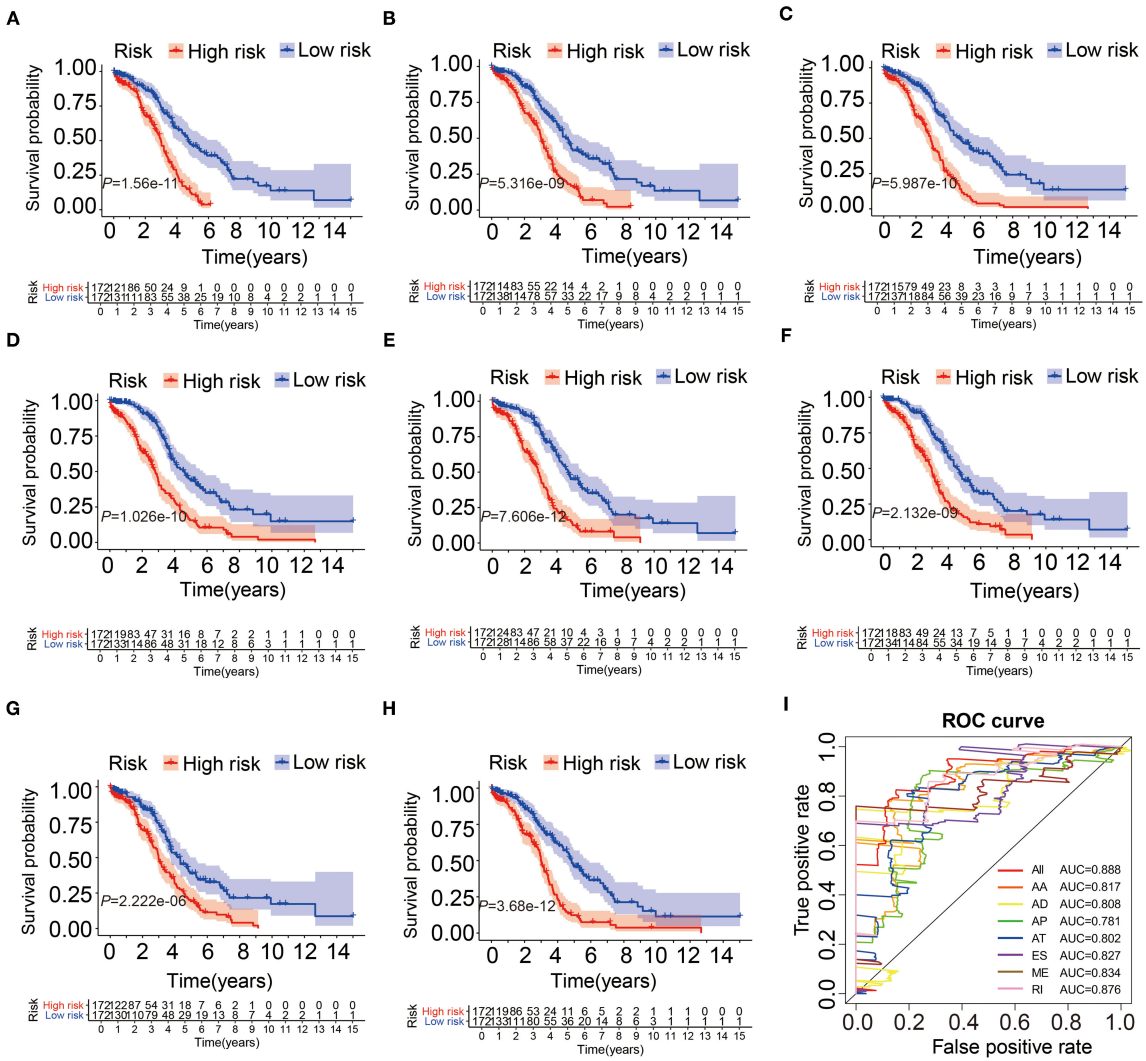
The Kaplan-Meier curves and ROC curves of prognostic AS models. **(A–G)** The Kaplan-Meier plots of seven types of AS events, respectively. **(H)** The Kaplan-Meier plots of combined prognostic model. **(I)** The ROC curves for overall survival of seven types of AS events and combined prognostic model.

We collected AS events related to prognosis by univariate Cox analysis and LASSO Cox regression analysis (Supplementary Figures 1A,B). Then, through multivariate Cox analysis, several prediction models based on these selected events were established. Finally, a joint prognostic model was established from different types of AS events (Supplementary Table 2). The Kaplan-Meier curve shown in Figures 3A–H indicated that the OS of OV patients in the high-risk group was significantly shorter than that in the low-risk group, suggesting that these AS signatures may be powerful bio-markers to distinguish the prognosis of OV patients. Obviously, the joint prognostic model showed better predictive performance than a single type of AS events (Figure 3H). In addition, to evaluate the significance of each gene in ovarian cancer, we conducted Kaplan-Meier analysis of each gene's mRNA level and found most of these genes had consistent prognostic value, shown in Supplementary Figure 2. In order to compare the predictive ability of these prognostic models, ROC analysis was performed. The results showed that all models had strong prediction performance, with AUC values ranging from 0.781 to 0.888 (Figure 3I). It was conceivable that an integrated prognostic model, rather than a single prognostic model, including different types of AS events, had the highest efficiency of 0.888 (AUC). The risk score, survival status and expression profile of all AS models were shown in Supplementary Figure 3.

### Validation of the AS Signatures in the Testing Cohort

**Figure 4:**
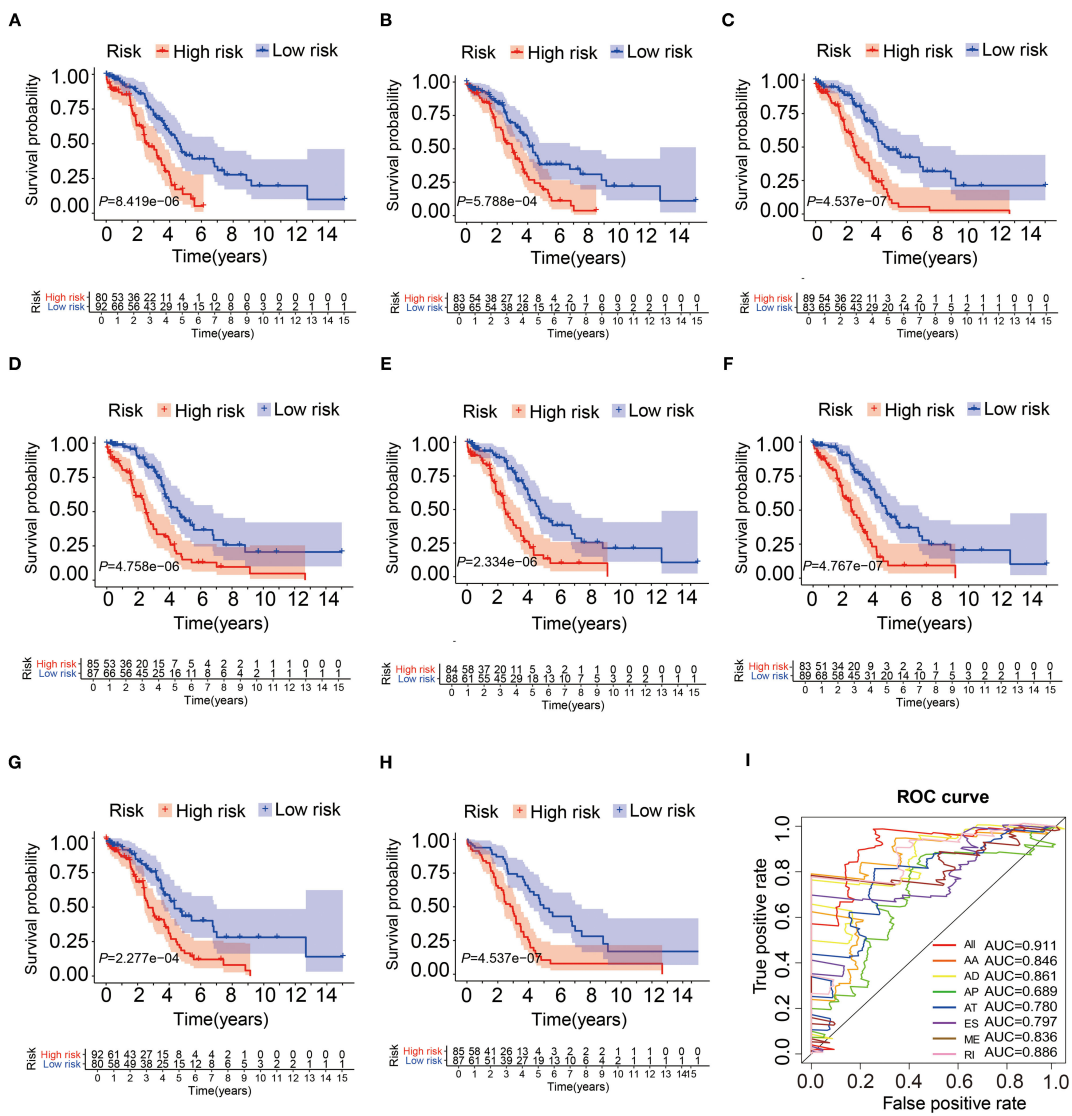
The Kaplan-Meier curves and ROC curves of prognostic AS models in the testing cohort. **(A–G)** The Kaplan-Meier plots of seven types of AS events, respectively. **(H)** The Kaplan-Meier plots of combined prognostic model. **(I)** The ROC curves for overall survival of seven types of AS events and combined prognostic model.

In order to verify the prognostic efficiency of AS signatures, we also performed Kaplan-Meier analysis and ROC curve (Figures 4A–I) in 172 patients (Supplementary Table 1) in the same model test cohort. The Kaplan-Meier curve showed that these seven AS events and comprehensive prognostic characteristics had strong ability to distinguish between favorable and unfavorable survival in patients with OV (Figures 4A–H). The previous article only obtained a single result through the overall analysis (18). However, this study validated the results by using inner testing cohort, which was a powerful evidence of this paper. Then, the AUC of all models ranged from 0.689 to 0.911, and the combined prognostic model had the highest AUC value, indicating that these prognostic AS signatures were more accurate (Figure 4I). These results suggested that these AS characteristics, especially the combined prognostic model, can be considered as powerful indicators for predicting the overall survival of patients with OV.

### AS Signatures Were Independent to Other Clinical Characteristics

**Figure 5:**
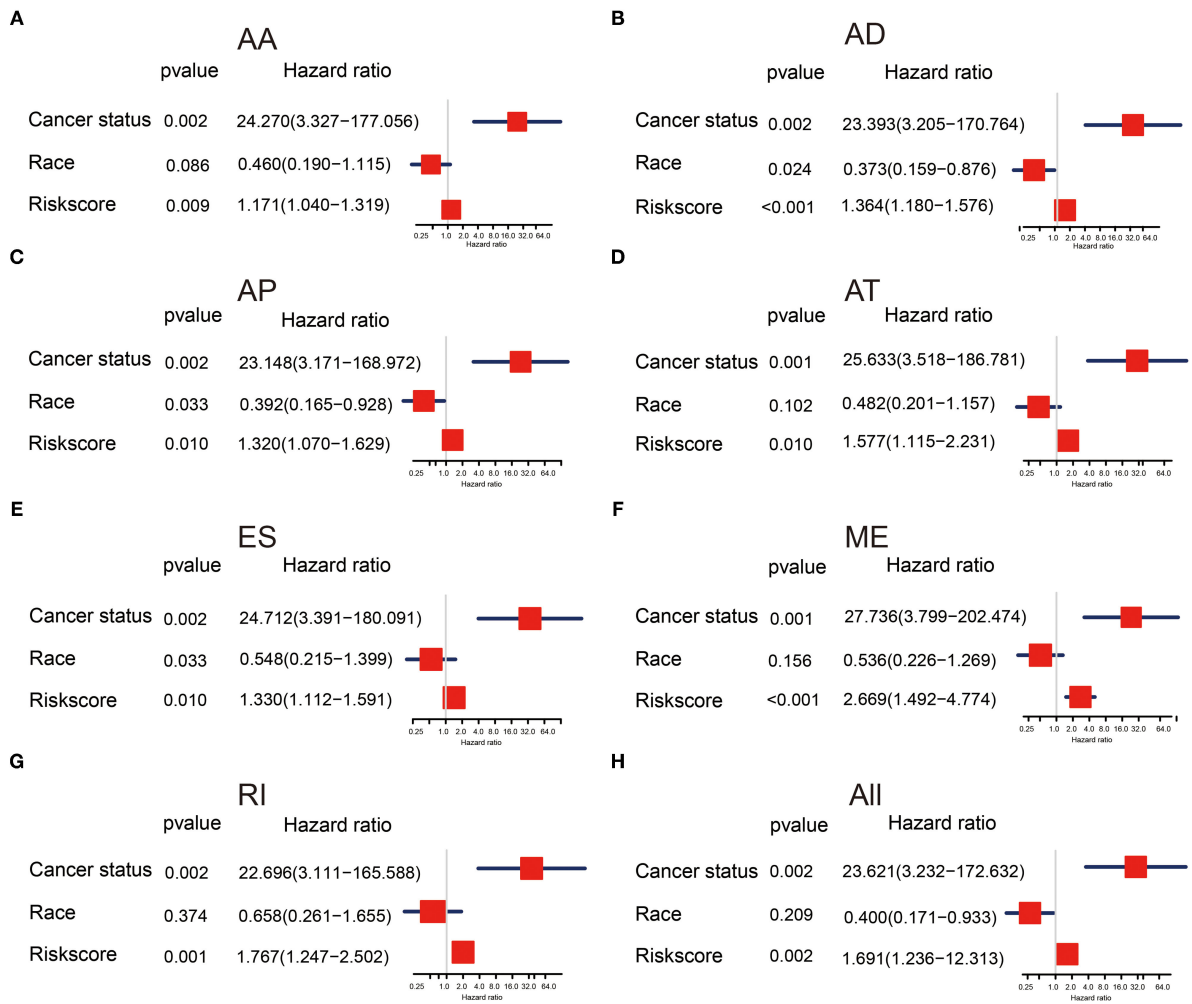
**(A–H)** Forest plots of hazard ratios of risk scores and clinical characteristics from multivariate Cox analyses.

To evaluate the effectiveness of AS signatures and other clinical features in predicting survival, we performed univariate and multivariate Cox analysis to determine whether these AS signatures can be used as independent risk factors for evaluating the prognosis of OV patients. Uni-variate Cox analysis showed that all risk scores, cancer status, and ethnicity were significantly correlated with overall survival (Supplementary Figure 4). In addition, multivariate Cox analysis showed that risk score and cancer status still had prognostic ability, suggesting that risk score and cancer status were independent prognostic factors for patients with OV (Figures 5A–H). In multivariate analysis, it was found that cancer status increased the risk of patients, which may provide a reference for clinicians and patients to choose treatment. Furthermore, it is well-known that Homologous recombination repair (HRR) pathway deficiency (HRD) is involved in the tumorigenesis and progression of high-grade serous ovarian carcinoma (20) as well as AS factors can contribute to the DNA damage response signaling (21). Therefore, we also analyzed the important of HRD in ovarian cancer and found the HRD signature had prognostic value in univariate Cox analysis, but HRD signature were not independent prognostic factors in multivariate Cox analysis (shown in Supplementary Figure 4). This result demonstrated that HRD may interactive with AS event, which is consistent with the Takaya's research (20). Taken together, these results showed that the characteristics of AS events had strong predictive effect in patients with OV.

### Prognostic AS and SFs Networks

**Figure 6:**
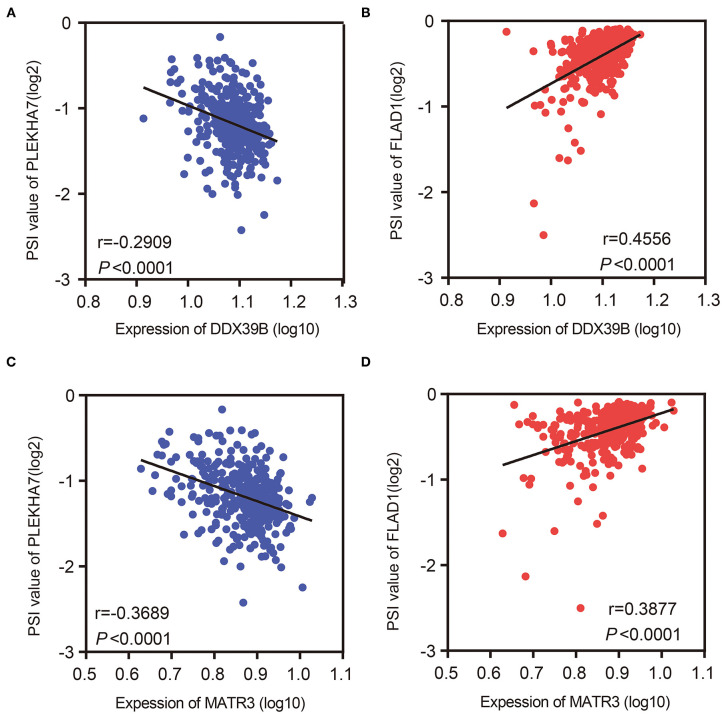
Correlations between expression of splicing factors and PSI values of AS events. **(A,B)** Representative dot plots of correlations between splicing factor DDX39B and AT of PLEKHA7 and AD of FLAD1, respectively. **(C,D)** Representative dot plots of correlations between splicing factor MATR3 and AT of PLEKHA7 and AD of FLAD1, respectively.

Splicing factor was a kind of RNA-binding protein, which could affect the exon selection and splicing site selection of pre-mRNA. Interestingly, in many types of cancer, widespread misalignment of AS events can easily be programmed by specific SFs. To identify specific SF that was closely related to AS events associated with prognosis in OV, we used the Spearman test to calculate the correlation coefficient between SFs and the most important prognostic AS events in patients with OV (Supplementary Figures 5A–E). Among these networks, 22 SFs (purple dots) were significantly associated with 249 prognosis-related AS events, involving 136 favorable AS events (green dots) and 113 adverse AS events (red dots). There was a positive correlation (red line) between the most favorable prognosis-related AS events (red dots) and SFs (purple dots), while most adverse prognosis-related AS events (green dots) were negatively correlated with SFs (green lines). For example, the expression of DDX39B and MATR3 was negatively correlated with the AT of PLEKHA7 (Figures 6A,C), but positively correlated with the AD of FLAD1 (Figures 6B,D).

### The AS Signature had Better Predictive Property Than mRNA Signature

**Figure 7:**
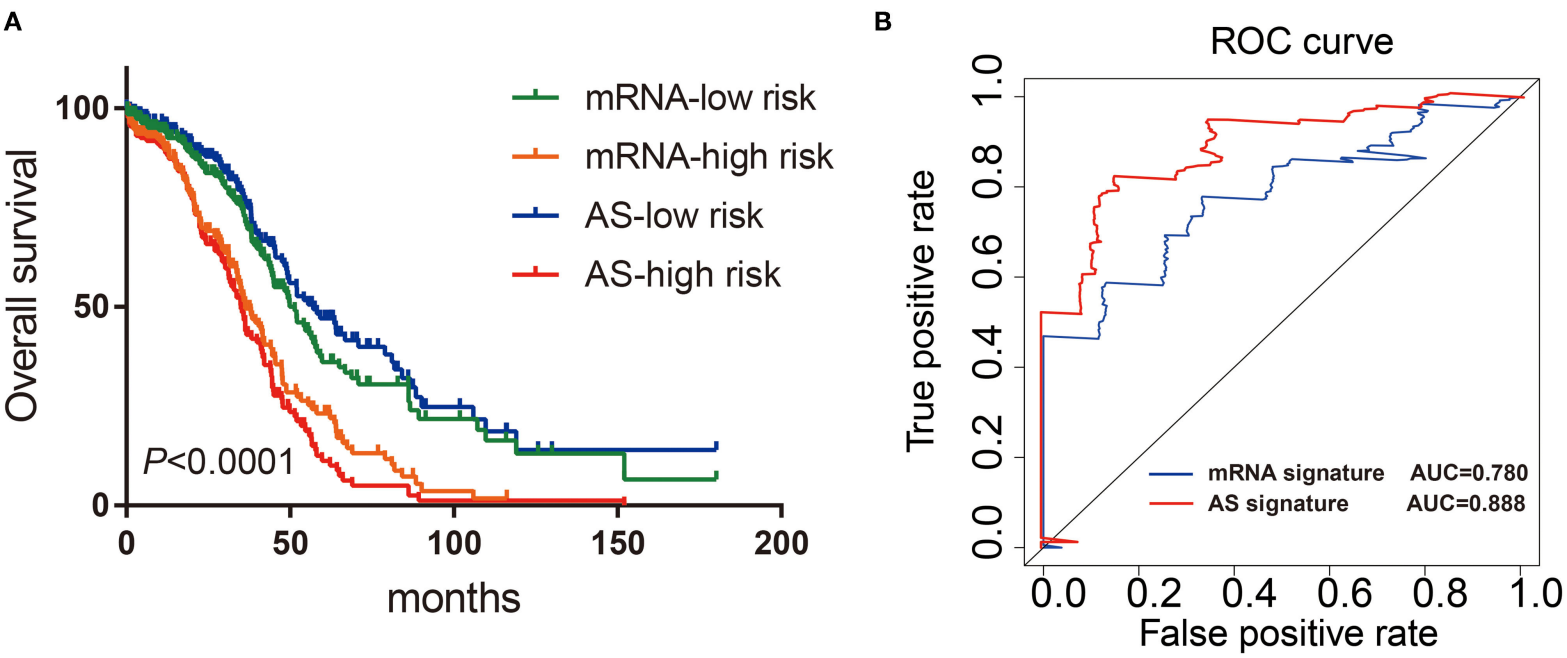
Comparison of Kaplan-Meier and time-dependent ROC analysis of AS signature with mRNA signature. **(A)** Comparison of Kaplan-Meier analysis of AS signature and mRNA signature. **(B)** Comparison of ROC analysis of the sensitivity and specificity of AS signature and mRNA signature.

Finally, we constructed the mRNA signature of OV patients through uni-variate and multivariate Cox analysis: mRNA risk score signature = (−2.1573^*^HMGB3) + (2.6940^*^PDS5B) + (1.9730^*^NBN) + (1.5767^*^CDKN1B) + (−2.1324^*^PRIM2) + (2.4335^*^CDKN2A). Then, the Kaplan-Meier and ROC curves were implemented to compare the prognostic ability between AS signatures and mRNA signatures. Both results from Kaplan-Meier and ROC analysis showed that AS signature had better survival rate and higher ROC than mRNA signature (Figures 7A,B). These data showed that the predictive ability of AS signature was better than that of mRNA signature. In general, AS signature could be used as a superior indicator to predict the prognosis of OV patients.

## Discussion

AS can not only play an important role in maintaining the normal physiological process of human body, but also a key mechanism leading to all kinds of pathology. In the past decade, many investigations have disclosed that AS events are detected in the occurrence and development of many human diseases, including tumors. In the field of AS research, recent studies have shown that several mutations in alternative splice and different splicing events in specific cancers may be used as indicators for the diagnosis, prediction, and prognosis of ovarian cancer. For example, the low expression of CADM1 in OV has been reported to inhibit the proliferation and migration of ovarian cancer cells through the PI3K/Akt/mTOR pathway (22). It has been proved that CLN3 is abnormally highly expressed in a variety of cancer-related cell lines, including ovarian cancer (23). Further studies have shown that CLN3 plays an important role in tumorigenesis and drug resistance of ovarian cancer (24). It is reported that the high expression of CTBP2 is closely related to the poor prognosis of patients with OV (25). A number of reports have shown that RAD51B variants are poor prognostic factors in ovarian cancer (26–29). In OV, the ES of TFCP2 is considered to be a favorable prognostic factor (18). All these studies show that the results of this paper are basically consistent with the previous reports. Overall, these reports remind us that further exploration of AS in OV may contribute to the discovery of some powerful diagnostic bio-markers and therapeutic targets.

In recent years, with the progress of high-throughput technology, great progress has been made in the identification of the most common genetic aberrations in splice sites and splice bodies. Therefore, the study of abnormal patterns of AS is very helpful for the development of new OV treatment strategies.

In this study, we screened out some regulatory splicing factors and AS events in ovarian cancer in order to further and comprehensively understand the variant RNA splicing pattern. Of the 1,125 host genes, a total of 1,429 AS events were significantly associated with the survival status of patients in OV. Interestingly, the top 20 AS events related to survival tend to have a good prognosis. Further analysis shows that the prognostic prediction model based on ES events is more efficient than the model based on six additional AS events to distinguish the survival of OV patients. In addition, based on the differential splicing patterns of 13 genes, an ideal prognostic model is proposed. This model has high performance in the risk stratification of OV patients and has great potential in predicting the prognosis of OV patients. All these results suggest that AS events have a wide range of variability in the tumor environment, and these changes can greatly affect the clinical outcome of cancer patients. Mutated RNA splicing related genes and their corresponding splicing regulatory genes enrich our understanding of AS and provide potential bio-markers and potential targets for prognosis in patients with OV. In addition, this paper also strengthens our opinion on finding more AS signatures related to prognosis in the OV cohort, which may help to significantly increase the life expectancy of highly personalized treatment according to different treatment responses of OV patients with different gene AS status and different AS levels based on the same gene.

The test cohort accounted for 50% of all patients and was selected randomly (30). By applying these AS signatures in the test cohort, significant risk stratification and high AUC values for patient survival were also observed, which proved the efficiency and rationality of LASSO regression analysis (19). The internal verification results can greatly increase the reliability and potential of clinical application. In addition, uni-variate and multivariate Cox analysis showed that AS signatures were independent risk factors for the prognosis of patients with OV. These results suggested that these AS signatures were potentially reliable bio-markers for predicting the survival of patients with OV.

It is reported that AS events are largely regulated by their corresponding core SFs. Therefore, we further exploded the relationship between these SFs expression levels and survival-related AS events in OV. 22SFs, including IGF2BP3, BAG2, and RNF213, were found to be associated with survival-related AS events. Interestingly, IGF2BP3 has been found to be associated with chemotherapy resistance and poor prognosis of ovarian cancer (31–33). BAG2 can promote the metastasis and proliferation of gastric cancer (34). By affecting the MAPK/JNK signal pathway (35), RNF213 can inhibit the carcinogenesis of glioblastoma. However, it is necessary to further explore the more specific regulatory mechanism of the AS-SF network.

At present, many studies have established the prognostic characteristics of cancer patients based on RNA expression. Therefore, we compared our AS signature with mRNA signature and found that AS signature has better prediction ability than mRNA signature. These data provided a useful evidence that AS signature can be used in clinical applications.

However, the limitations should be acknowledged for this study. First, the prognostic AS signature were identified by reasonable and reliable statistical approaches, but the results was only verified in TCGA database and simple size is small. Second, the TCGA database represents part but not all of the possible clinical characteristics information, such as alcohol consumption and social status were not available in TCGA database. Thus, we could not control those factors that might cause biases in our analysis. Finally, we identified several prognostic AS events can be regulated by some key SFs. Unfortunately, the study of the specific mechanism between AS events and key SFs is unclear and experimental studies on these mechanisms are greatly needed to further understand their functional role in OC.

In summary, this study revealed the prognostic value of several AS events in TCGA-OV, and these prognostic AS events can be regulated by some key SFs. Our findings may provide new prospects for effective treatment of AS events in patients with OV.

## Data Availability Statement

The datasets presented in this study can be found in online repositories. The names of the repository/repositories and accession number(s) can be found in the article/Supplementary Material.

## Author Contributions

JL, RW, and XL designed the study and wrote the paper. JL and DL performed research. JL, DL, XW, and RW analyzed data. JL and XL wrote the first draft of the paper and edited the manuscript. All authors have read and approved the final version of the manuscript.

## Conflict of Interest

The authors declare that the research was conducted in the absence of any commercial or financial relationships that could be construed as a potential conflict of interest.

## Supplementary Material

Supplementary Figure 1 Survival-related AS events were selected using the LASSO Cox analysis. **(A)** LASSO coefficient profiles of the candidate survival-related AS events. **(B)** Dotted vertical lines were drawn at the optimal values by using the minimum criteria.

Supplementary Figure 2 The Kaplan-Meier curves of each prognostic AS' mRNA level.

Supplementary Figure 3 **(A–H)** Construction and analysis of risk scores of combined prognostic model. The top panels indicate the risk scores of the patients. The middle panels depict the survival status and survival time of patients distributed by risk score. The bottom panels display the heatmap of the PSI values for predictive factors distributed by risk score.

Supplementary Figure 4 Forest plots of hazard ratios of risk scores and clinical characteristics from univariate and multivariate Cox analyses.

Supplementary Figure 5 Correlation network between the expression of survival splicing factors and the PSI values of AS genes produced by Cytoscape. Purple dots were survival-related splicing factor. Green/Red dots were favorable/adverse AS events. Red/Green lines represent positive/negative correlations between substances.

Supplementary Table 1 Clinical characteristics of OV in TCGA data set.

Supplementary Table 2 OV-specific genes involved in combined prognostic model.OV, ovarian cancer; HR, hazard ratio.
