# Supplementary figures and images for: Systematic Profiling of Alternative Splicing Events in Ovarian Cancer
Source: Front Oncol. 2021 Mar 8;11:622805. doi: 10.3389/fonc.2021.622805 (PMC7982604; doi:10.3389/fonc.2021.622805)

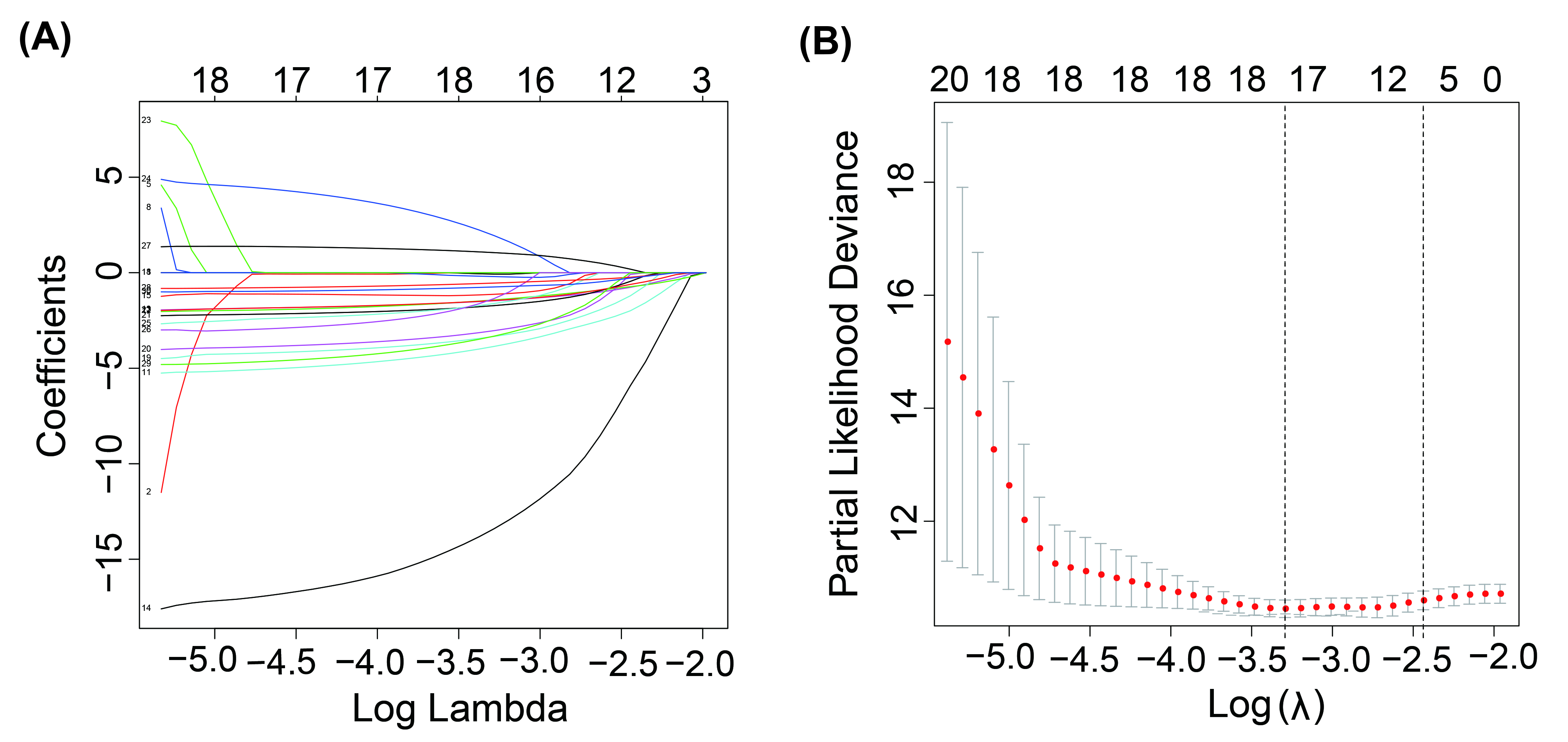

Supplement: Supplementary Figure 1 — Survival-related AS events were selected using the LASSO Cox analysis. (A) LASSO coefficient profiles of the candidate survival-related AS events. (B) Dotted vertical lines were drawn at the optimal values by using the minimum criteria. [file Image_1.TIF]

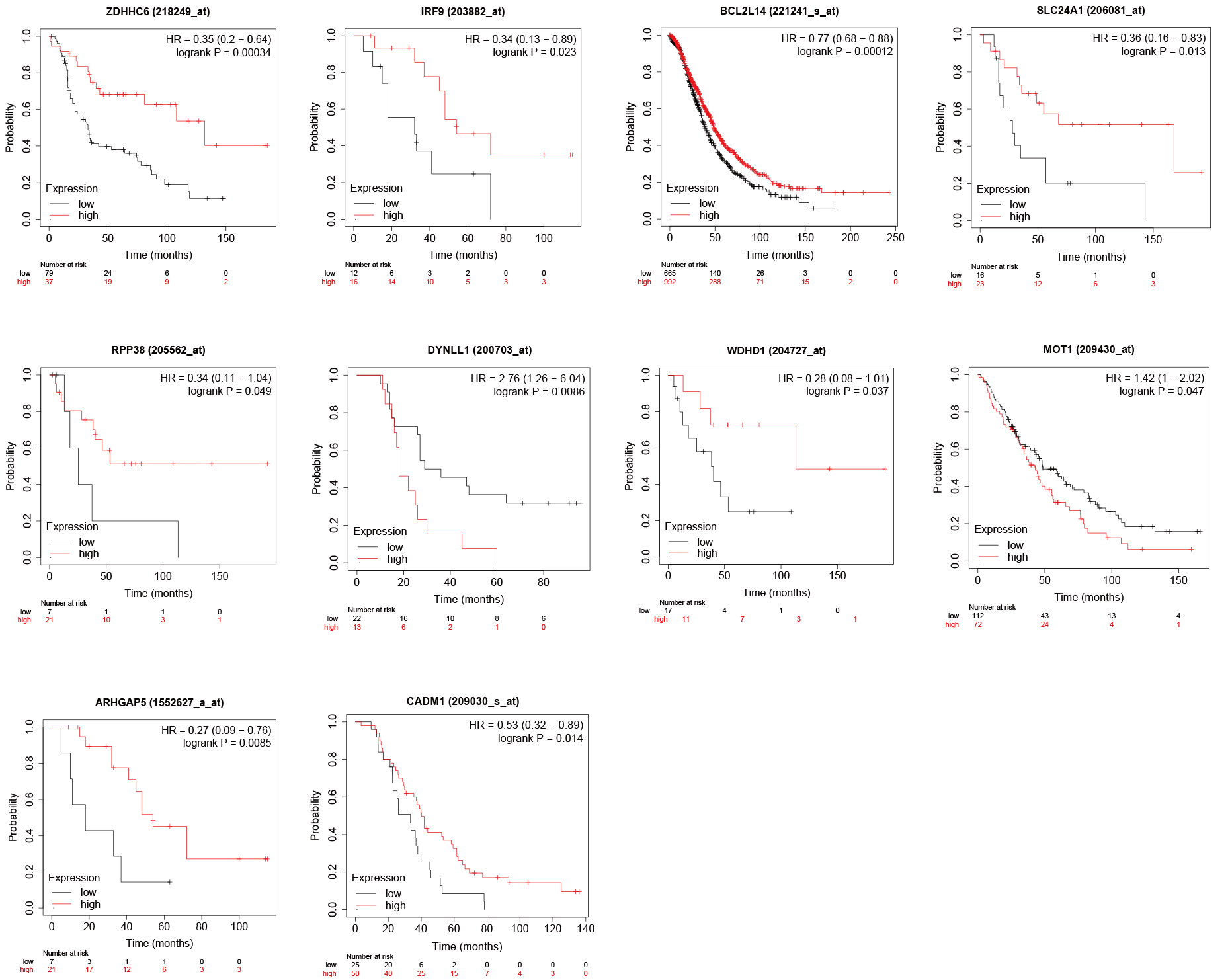

Supplement: Supplementary Figure 2 — The Kaplan-Meier curves of each prognostic AS' mRNA level. [file Image_2.TIF]

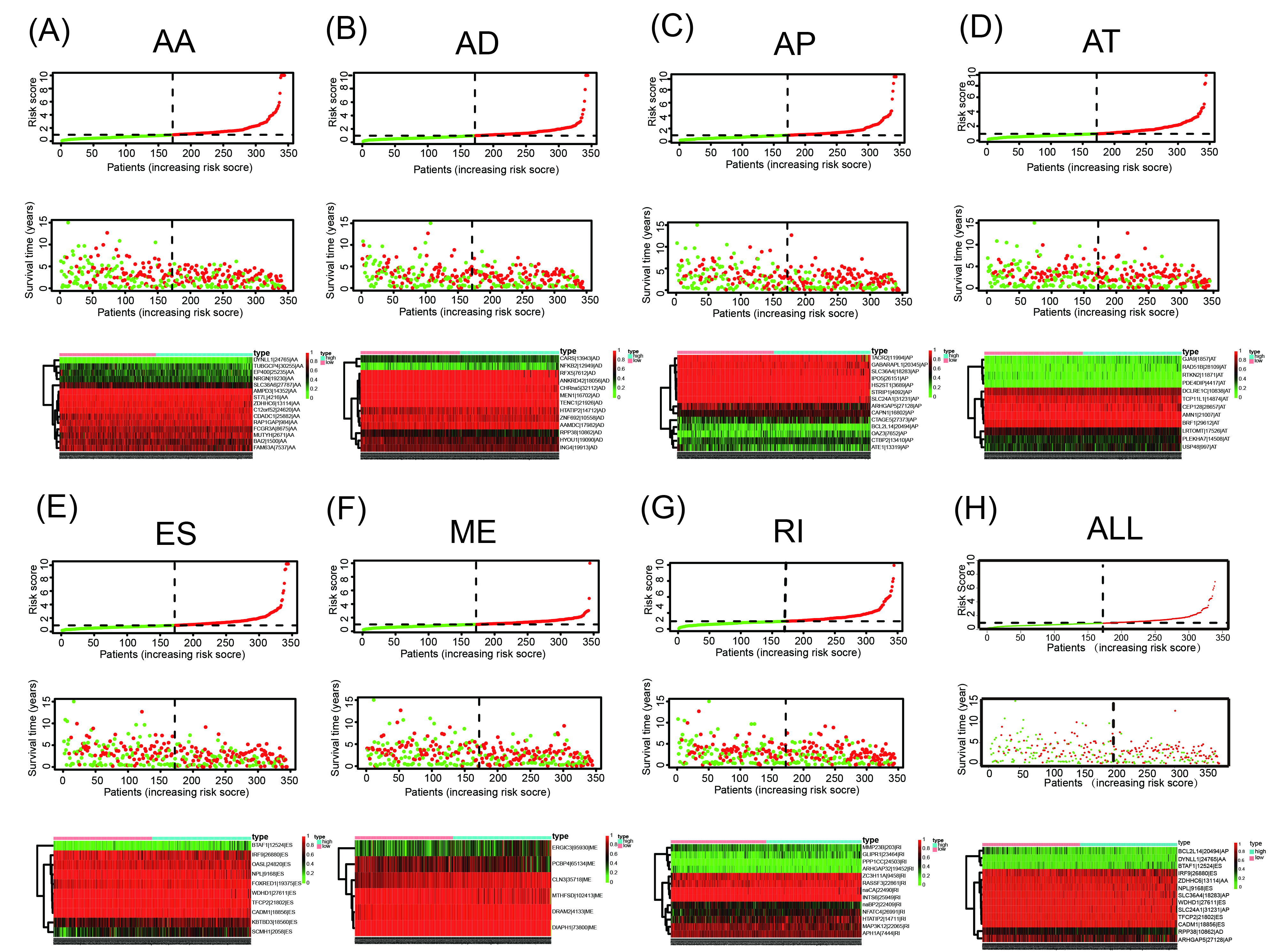

Supplement: Supplementary Figure 3 — (A–H) Construction and analysis of risk scores of combined prognostic model. The top panels indicate the risk scores of the patients. The middle panels depict the survival status and survival time of patients distributed by risk score. The bottom panels display the heatmap of the PSI values for predictive factors distributed by risk score. [file Image_3.TIF]

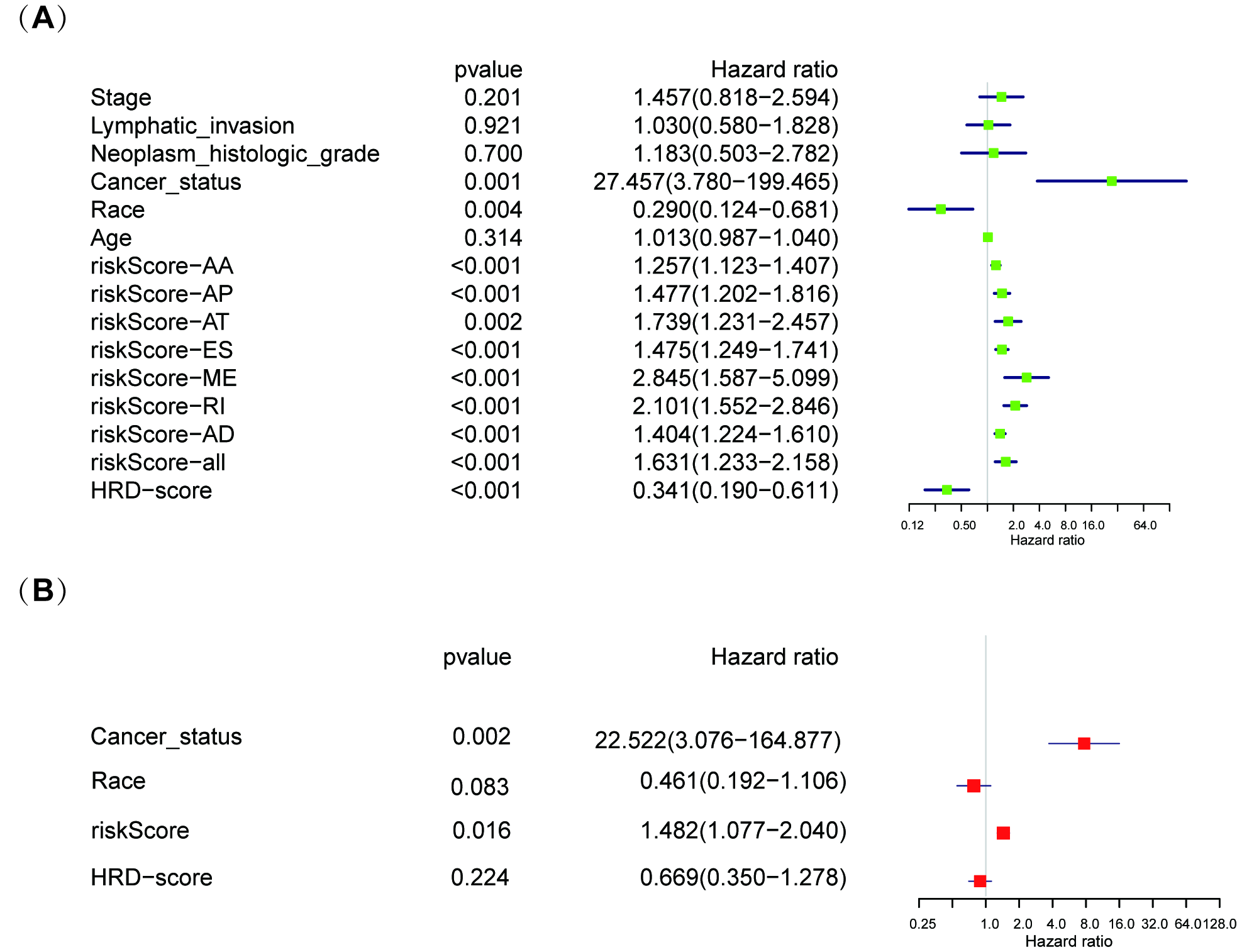

Supplement: Supplementary Figure 4 — Forest plots of hazard ratios of risk scores and clinical characteristics from univariate and multivariate Cox analyses. [file Image_4.TIF]

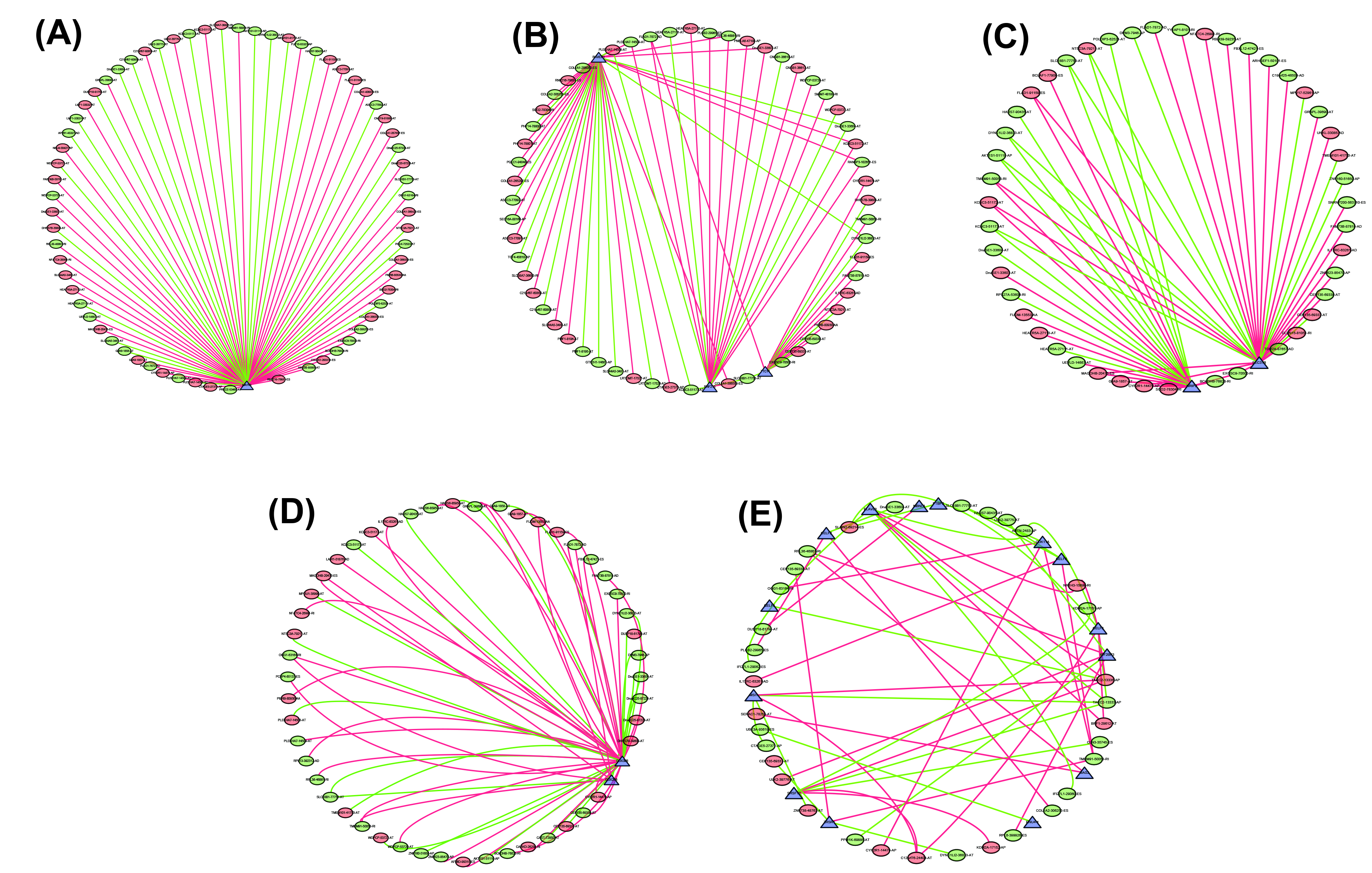

Supplement: Supplementary Figure 5 — Correlation network between the expression of survival splicing factors and the PSI values of AS genes produced by Cytoscape. Purple dots were survival-related splicing factor. Green/Red dots were favorable/adverse AS events. Red/Green lines represent positive/negative correlations between substances. [file Image_5.TIF]
