# Supplementary material for: Systematic Profiling of Alternative Splicing Events in Ovarian Cancer
Source: Front Oncol. 2021 Mar 8;11:622805. doi: 10.3389/fonc.2021.622805 (PMC7982604; doi:10.3389/fonc.2021.622805)
Supplement: Supplementary Table 1 — Clinical characteristics of OV in TCGA data set. [file Table_1.DOCX]

Table S1. Clinical characteristics of OV in TCGA data set

| Characteristics | Training series (%) | Testing cohort (%) |
| --- | --- | --- |
| Age(years) |  |  |
| >65 | 112/344 (32.56) | 58/172 (33.72) |
| ≤65 | 232/344 (67.44) | 114/172 (66.28) |
| Clinical stage |  |  |
| I | 1/344 (0.29) | 0/172 (0) |
| II | 20/344 (5.81) | 15/172 (8.72) |
| III | 267/344 (77.62) | 134/172 (77.91) |
| IV | 54/344 (15.70) | 23/172 (13.37) |
| Race |  |  |
| Non-white | 34/344 (9.88) | 21/172 (12.21) |
| White | 300/344 (87.21) | 146/172 (84.88) |
| Lymphatic invasion |  |  |
| NO | 43/344 (12.50) | 27/172 (15.70) |
| YES | 91/344 (26..45) | 57/172 (33.14) |
| Grade |  |  |
| G1 | 1/344 (0.29) | 0/172 (0) |
| G2 | 41/344 (11.92) | 18/172 (10.47) |
| G3 | 292/344 (84.88) | 151/172 (87.79) |
| G4 | 1/344 (0.29) | 1/172 (0.58) |
| Cancer status |  |  |
| Tumor free | 79/344 (22.97) | 42/172 (24.42) |
| With tumor | 221/344 (64.24) | 106/172 (61.63) |
