# Supplementary material for: Systematic Profiling of Alternative Splicing Events in Ovarian Cancer
Source: Front Oncol. 2021 Mar 8;11:622805. doi: 10.3389/fonc.2021.622805 (PMC7982604; doi:10.3389/fonc.2021.622805)
Supplement: Supplementary Table 2 — OV-specific genes involved in combined prognostic model.OV, ovarian cancer; HR, hazard ratio. [file Table_2.DOCX]

Table S2. OV-specific genes involved in combined prognostic model.

| Gene | coefficient | HR | Lower95 | Upper95 | *P* value | Type |
| --- | --- | --- | --- | --- | --- | --- |
| ZDHHC6 | -5.4683 | 0.0042 | 9.81E-05 | 0.1813 | 0.0043 | AA |
| IRF9 | -2.0030 | 0.1348 | 0.0373 | 0.4863 | 0.0022 | ES |
| TFCP2 | -18.7000 | 7.54E-09 | 1.49E-12 | 3.82E-05 | 1.73E-05 | ES |
| BCL2L14 | -2.1710 | 0.1140 | 0.0241 | 0.5379 | 0.0060 | AP |
| SLC36A4 | -4.8649 | 0.0077 | 0.0008 | 0.0698 | 1.5E-05 | AP |
| SLC24A1 | -4.1770 | 0.0153 | 0.0012 | 0.1889 | 0.0011 | AP |
| NPL | -2.5550 | 0.0776 | 0.0080 | 0.7526 | 0.0274 | ES |
| RPP38 | -2.1867 | 0.1122 | 0.0235 | 0.5352 | 0.0060 | AD |
| DYNLL1 | 5.9958 | 4.0173 | 3.1311 | 5.0675 | 0.0102 | AA |
| WDHD1 | -3.2646 | 0.0382 | 0.0017 | 0.8194 | 0.0368 | ES |
| BTAF1 | 1.4711 | 4.3544 | 0.7446 | 25.4620 | 0.1025 | ES |
| ARHGAP5 | -0.9586 | 0.3833 | 0.1470 | 0.9994 | 0.0498 | AP |
| CADM1 | -5.1570 | 0.0057 | 0.0003 | 0.1033 | 0.0004 | ES |

OV, ovarian cancer; HR, hazard ratio
